# Inflammatory Endotypes and Microbial Associations in Chronic Rhinosinusitis

**DOI:** 10.3389/fimmu.2018.02065

**Published:** 2018-09-19

**Authors:** Michael Hoggard, Sharon Waldvogel-Thurlow, Melissa Zoing, Kevin Chang, Fiona J. Radcliff, Brett Wagner Mackenzie, Kristi Biswas, Richard G. Douglas, Michael W. Taylor

**Affiliations:** ^1^School of Biological Sciences, The University of Auckland, Auckland, New Zealand; ^2^School of Medicine, The University of Auckland, Auckland, New Zealand; ^3^Department of Statistics, Statistical Consulting Centre, The University of Auckland, Auckland, New Zealand; ^4^School of Medical Sciences, The University of Auckland, Auckland, New Zealand; ^5^Maurice Wilkins Centre for Molecular Biodiscovery, The University of Auckland, Auckland, New Zealand

**Keywords:** inflammation, phenotypes, endotypes, cluster analysis, microbiota, dysbiosis, chronic rhinosinusitis

## Abstract

A complex mix of inflammatory and microbial associations underscores the chronic inflammatory condition chronic rhinosinusitis (CRS), and the etiology remains poorly understood. Recent work has begun to delineate between variants (endotypes) of CRS on the basis of inflammatory biomarkers. This study aimed to assess inflammatory patterns in CRS phenotypes, identify putative endotypes of CRS, and to assess inflammatory associations with the sinonasal microbiota. Ten cytokines and six inflammatory cell types were assessed in mucosal biopsies from 93 CRS subjects and 17 controls via cytometric bead array and immunohistochemical techniques. Putative endotypes were identified via cluster analysis of subjects on the basis of inflammatory markers and comorbidities including polyposis, asthma, and aspirin sensitivity. Finally, previously published bacterial data for this cohort were reanalyzed to evaluate associations with inflammatory markers and CRS subtypes. Inflammatory patterns were highly variable within standard CRS phenotypes. Cluster analysis identified eight subject clusters, with strong delineation on the basis of polyposis and asthma, but also subtle distinctions in inflammatory markers. An association was also identified between depletion of several “health-associated” bacterial taxa, reduced bacterial diversity and increased overall bacterial load, with markers of inflammation and clinical severity. This study contributes to ongoing efforts to define distinct endotypes of CRS on the basis of underlying inflammatory processes, and also offers compelling evidence of a link between bacterial community dysbiosis and inflammation in CRS. Further resolving the heterogeneity of CRS is vital to inform clinical management and personalized treatment approaches.

## Introduction

Chronic rhinosinusitis (CRS) is a group of debilitating chronic inflammatory conditions affecting the upper airways in up to 5% of the population (1, 2). It represents a considerable burden on patient quality of life and healthcare systems alike (1–3). As with previous developments in the understanding of lower respiratory inflammatory conditions such as asthma (4, 5), CRS is increasingly understood as an umbrella term covering a diverse array of chronic inflammatory subtypes (6–9).

Recent studies have made initial steps in characterizing CRS in terms of variants of the underlying inflammatory processes. One study identified 10 putative subtypes of CRS on the basis of a suite of inflammatory markers in a large cohort of 262 subjects (173 CRS subjects compared against results from 89 control subjects) (10). Inflammatory markers included the cytokines interleukin- (IL-)1β, IL-5, IL-6, IL-8, IL-17A, tumor necrosis factor- (TNF-)α, interferon- (IFN-)γ, and transforming growth factor- (TGF-)β1, as well as other inflammatory biomarkers such as myeloperoxidase, eosinophilic cationic protein, IgE, and *Staphylococcus aureus* enterotoxin-specific (SE)-IgE. While standard phenotypic divisions (on the basis of associated nasal polyposis) fell largely into separate inflammatory groups, multiple inflammatory subtypes were identified. A study of large-scale gene expression patterns in CRS similarly illustrated the heterogeneous nature of inflammation within CRS phenotypic subtypes (11). Most recently, an early indication of a link between the inflammatory heterogeneity of CRS and the associated sinonasal bacterial community patterns was also observed (12).

This study builds on the aforementioned work, assessing an array of inflammatory cells and signaling markers (CD3^+^ T cells, CD20^+^ B cells, CD68^+^ macrophages, plasma cells, eosinophils, neutrophils, IL-2, IL-4, IL-5, IL-6, IL-8, IL-10, IL-13, IL-17A, IFN-γ, and TNF), together with the presence of comorbidities (including polyposis, asthma, and a history of aspirin sensitivity), in a cohort of 110 subjects, to delineate between inflammatory variants that might be suggestive of endotypes in this condition. Additionally, a reanalysis of previously published bacterial microbiota data (13) for these subjects (collected at the same intraoperative time point) was performed. We investigated the extent to which inflammatory endotypes might better describe the observed patterns in bacterial communities when compared to traditional phenotypes based on polyposis or asthma, as well as more general associations between patterns in bacterial communities and inflammatory types underlying CRS.

## Materials and methods

### Patient recruitment and sample collection

One hundred and ten subjects were recruited for this study, including 93 subjects undergoing endoscopic sinus surgery for extensive bilateral CRS, and 17 controls undergoing an endoscopic procedure for indications other than CRS (predominantly pituitary adenoma surgery and dacrocystorhinostomy). CRS subjects were selected as per the 2012 European Position Paper guidelines (1). Exclusion criteria for recruitment included sinonasal vasculitis, known immunodeficiency, and age less than 18 years. At the time of recruitment, patient demographics, symptom severity scores [based on a rating of five nasal symptoms, as previously described (13–15)], and Lund-Mackay scores were collected. Demographics data included age, gender, ethnicity, revision surgery for CRS, asthma status, a history of aspirin sensitivity, and antibiotic and corticosteroid administration in the 4 weeks prior to surgery. Sampling was conducted at the time of surgery, before the administration of topical mucosal vasoconstrictors.

For each subject, two tissue biopsies were collected from the bulla ethmoidalis using a Blakesley forcep, rinsed in sterile saline, placed in sterile 2 mL collection tubes on ice, and transported to the laboratory within 2 h of collection. Tissue intended for histological analysis was fixed in Carnoy's solution (60% ethanol, 30% chloroform, 10% glacial acetic acid), before being placed in 70% ethanol prior to paraffin embedding. Tissue samples intended for cytokine analysis were fixed in Ambion RNA*later* (Life Technologies, Auckland, New Zealand) and stored at −20°C until the time of sample processing.

This study was approved by the New Zealand Health and Disability Ethics Committee (NTX/08/12/126), and written informed consent was obtained from all participants.

### Histology

Tissue sections of 4 μm thickness were prepared from paraffin-embedded tissue. Batches of sections were processed separately for each of the following: (1) Eosinophil and neutrophil cell counts (hematoxylin-eosin (H&E) staining as per routine practice); (2) Plasma cell counts [methyl green-pyronin staining as previously described (16)]; (3) CD3^+^ T cells [anti-CD3 monoclonal antibody (mAb)]; (4) CD20^+^ B cells (anti-CD20 mAb); and (5) CD68^+^ macrophages (anti-CD68 mAb) (Supplementary Figure 1). Immunohistochemistry to enumerate CD3^+^ T cells, CD20^+^ B cells, and CD68^+^ macrophages was conducted following antigen retrieval using a pressurized heat-retrieval method (2100 retriever) with citrate buffer (pH 6). Sections were incubated with either mouse anti-CD3 (clone LN10), anti-CD20 (clone L26), or anti-CD68 (clone 514H12) (Leica Biosystems, Newcastle Upon Tyne, UK) and Novocastra IHC Diluent (Leica Biosystems, Newcastle Upon Tyne, UK) at the following dilutions: 1:400; 1:200; and 1:50. Sections were then processed with the Novolink Polymer Detection System Kit (Leica Biosystems, Newcastle Upon Tyne, UK) as per the manufacturer's instructions. Negative controls (omitting the antibody) were included for all samples. Five representative high-power field images (63× magnification) were taken for each section using an epifluorescence Leica DMR upright microscope (Leica Microsystems, Wetzlar, Germany) with a SPOT camera (Diagnostic Instruments, MI) and analysis FIVE software (Olympus, Japan). Cell counts were conducted independently by two separate observers who were blinded to subject diagnoses at the time of counting. Cells were counted using ImageJ (NIH, Bethesda), with replicates for each cell type averaged for subsequent analyses.

### Cytokines analysis

Key human inflammatory cytokines were assessed within mucosal biopsies via Cytometric Bead Array as previously described (17). IL-2, IL-4, IL-6, IL-10, IL-17A, TNF, and IFN-γ were assessed using a Human T_h_1/T_h_2/T_h_17 Cytokine Kit (BD Biosciences). Flex set kits were also selected for an additional three cytokines of interest (IL-5, IL-8, and IL-13). In brief, tissue biopsies were first weighed and then added to lysis tubes with 2 mm ceramic beads and 200 μL PBS, and ruptured in a bead ruptor 24 (Omni International) at 3.55 m/s for two cycles of 10 s. The homogenate was centrifuged at 1,500 × g for 5 min, and then 25 μL of the supernatant was transferred to assay tubes. Cytokine analysis on the supernatant was conducted as per the manufacturer's instructions. Data were acquired on an LSR II with BD FACSDiva Software (version 6.1.3) and analyzed using FCAP Array^TM^ (version 1.0.1) (BD Biosciences). A 5-parameter logistic model was used to produce standard curves with a fitting accuracy of >99.9% for all cytokines. Values below the threshold for the limit of detection for each cytokine were recorded as 0. IL-13 was below the limit of detection for all samples and was excluded from subsequent analyses. Final concentrations were normalized to concentration per gram of tissue based on the initial tissue weight.

### Bacterial analyses

Previously published bacterial data (including 16S rRNA gene amplicon-based assessment of community structure, and quantitative (q)PCR-based estimation of total bacterial load) from sample collections conducted concurrently with sampling for this study were available for 101 subjects from this cohort (13), and were re-analyzed to assess relationships with inflammatory markers and identified subject clusters. A table of bacterial OTUs and relative sequence abundance, genus-level taxonomic assignment of OTUs, and a Bray-Curtis dissimilarity matrix (as a measure of inter-subject bacterial community dissimilarity), were generated as previously described (13). Taxonomic summary plots and non-metric multidimensional scaling plots were generated in R (version 3.3.0) (18). To assess the main patterns in community composition, the 15 bacterial OTUs with the highest relative abundance overall were identified for subsequent analyses {collectively representing, on average, over 80% of composition of the bacterial communities (median [interquartile range] = 83% [71–95%])}, with all other OTUs resolved under “other.”

### Data analyses

Investigation of relationships between inflammatory variables, and clustering analysis of subjects to identify putative endotypes of CRS, were conducted as previously described (10). In brief, inflammatory variables were first analyzed using PCA and ascendant hierarchical clustering. PCA was followed by orthogonal rotation and Kaiser normalization, and variables with loadings < 0.4 were omitted. Hierarchical clustering incorporated correlation ratios and mixed PCA, with six clusters chosen as the optimal number on the basis of the Rand statistic (Supplementary Figure 2).

Subject clustering to identify inflammatory variants was then conducted. A Gower dissimilarity matrix was calculated in order to incorporate both categorical and continuous variables, followed by cluster analysis of subjects via the Partitioning Around Medoids method. With the exception of IL-13, all inflammatory variables measured were included in the cluster analysis. In addition, the clinical parameters (presence/absence) of polyposis, asthma, and aspirin sensitivity status were also included due to their widely accepted clinical importance in CRS. In order to assess inflammatory profiles in CRS relative to patterns in health, both controls and CRS subjects were included together in the subject clustering analysis. As previously described (10), the optimal cluster number was selected after assessing several parameters: a scree plot of mean silhouette width, the Baker-Huber Gamma statistic, the Hubert-Levin C index, Jaccard stability after bootstrap resampling, and plotting of subjects grouping based on the top two principal components (Supplementary Figure 3). Eight subject clusters were selected as the optimal differentiation of subjects, and were used for subsequent analyses.

PCA, hierarchical clustering, cluster index plots, subject clustering, and a heat map of the variables across the eight identified subject clusters were conducted and generated in R (18) using the packages cluster (19), ClustOfVar (20), clusterSim (21).

Demographics, inflammatory, and bacterial variables were compared between: A. standard clinical CRS phenotypes [controls, CRS without nasal polyps (CRSsNP), CRS with nasal polyps (CRSwNP), CRS with cystic fibrosis (CRSwCF)]; and B. the eight identified subject clusters. Differences between groups were first tested using Fisher's exact test (categorical variables) and the Kruskal-Wallis test (continuous variables). Pairwise comparisons were subsequently conducted for significant variables using Fisher's exact test (categorical variables) and Dunn's test of multiple comparisons (22) (continuous variables), with Bonferroni adjustment for multiple comparisons (23). Significance tests were 2-sided with α = 0.05. To further investigate whether patterns of bacterial colonization (presence) were characteristic of distinct subject clusters, predictive probabilities of the presence or absence of bacterial OTUs in each subject cluster were generated in R via logistic regression modeling (taking the binary response of the presence or absence of OTUs as the outcome). Analyses of deviance were conducted to test for significant differences in the presence/absence of OTUs across the subject clusters.

A heat map of relative sequence abundance patterns of the top 15 bacterial OTUs was generated in R. Ordering of subjects (rows) and bacterial OTUs (columns) was determined using average linkage hierarchical clustering of bacterial community similarity (based on Bray-Curtis dissimilarity), and co-occurrence patterns of bacterial OTUs, respectively.

Associations between variables were assessed via Spearman correlation analysis in R, with significance testing also conducted (α = 0.05) for each pairwise correlation test. The following variables were included in the analysis: inflammatory variables, all bacterial OTUs, bacterial community alpha diversity (Shannon index), bacterial load, symptom severity scores, and Lund-Mackay scores. A heat map of key variables identified in the Spearman correlation analysis was generated in R.

### Data availability

The demographics and inflammatory data generated and analyzed during this study are provided as an online supplementary file (Supplementary Data). The bacterial data (raw sequences) analyzed during this study were previously published (13) and are publicly available in the SRA-NCBI repository (accession SRP092370).

## Results

### Standard clinical phenotypes

#### Demographics

CRS with nasal polyps (CRSwNP) subjects were significantly more often asthmatic than CRS without nasal polyps (CRSsNP) and control subjects (*p*-values < 0.04), significantly more often had aspirin sensitivity and aspirin-exacerbated respiratory disease (AERD; asthma together with concomitant aspirin sensitivity), and had higher Lund-Mackay scores than CRSsNP subjects (*p-*values < 0.01) (Table 1). CRS with cystic fibrosis (CRSwCF) patients were significantly more often undergoing revision surgery for CRS than CRSsNP and CRSwNP patients (Supplementary Table 1). There were no significant differences in pairwise testing with correction for multiple comparisons between any groups as to whether subjects had taken antibiotics or steroids in the 4 weeks prior to surgery (Supplementary Table 1).

**Table 1:** Subjects' demographics.

	**Subject groups**				
**Variables^a^**	**Controls (*n* = 17)^b^**	**CRSsNP (*n* = 46)^b^**	**CRSwNP (*n* = 40)^b^**	**CRSwCF (*n* = 7)^b^**	**Unadjusted test *p-*value^c^**
Age	53 (23–84)	47 (19–67)	48 (18–71)	31 (21–50)	**0.010**
European	11/17 (65%)	37/46 (80%)	31/38 (82%)	6/7 (86%)	0.514
Gender	12/17 (71%)	24/46 (52%)	14/39 (36%)	5/7 (71%)	0.062
Asthma	1/17 (6%)	18/46 (39%)	28/40 (70%)	1/7 (14%)	<**0.001**
Aspirin sensitivity	1/17 (6%)	1/46 (2.2%)	12/40 (30%)	0/7 (0%)	**0.001**
AERD	0/17 (0%)	0/46 (0%)	11/40 (28%)	0/7 (0%)	<**0.001**
Preoperative antibiotics^d^	1/17 (6%)	7/46 (15%)	4/39 (10%)	4/7 (57%)	**0.024**
Preoperative corticosteroids^d^	0/17 (0%)	7/46 (15%	4/39 (10%)	2/7 (29%)	0.154
Revision surgery	NA	17/46 (37%)	18/39 (46%)	7/7 (100%)	**0.006**
Total symptom score	NA	14 (2–25)	18 (5 to 25)	18 (7–21)	0.084
Lund-Mackay score	NA	14 (5–21)	19 (5 to 24)	19 (12–24)	**0.001**

a *Categorical variables are summarized as proportion yes/total (%), except for gender, which is given as proportion female. Continuous variables are summarized as median (range)*.

b *Total cohort numbers for each group are given. The differences in total numbers for each variable reflect missing data for some subjects*.

c Difference between groups tested using Fisher's exact test for categorical variables and the Kruskal-Wallis test for continuous variables. Significant p-values (α = 0.05) are expressed in bold.

d *Antibiotics and/or steroids in the 4 weeks prior to surgery*.

*AERD, aspirin-exacerbated respiratory disease (asthma with concomitant aspirin hypersensitivity); CRS, chronic rhinosinusitis; CRSsNP, CRS without nasal polyps; CRSwNP, CRS with nasal polyps; CRSwCF, CRS with cystic fibrosis; NA, not applicable. Revision surgery = whether patients had previously undergone surgery for CRS prior to the current appointment*.

#### Inflammatory variables

**Figure 1:**
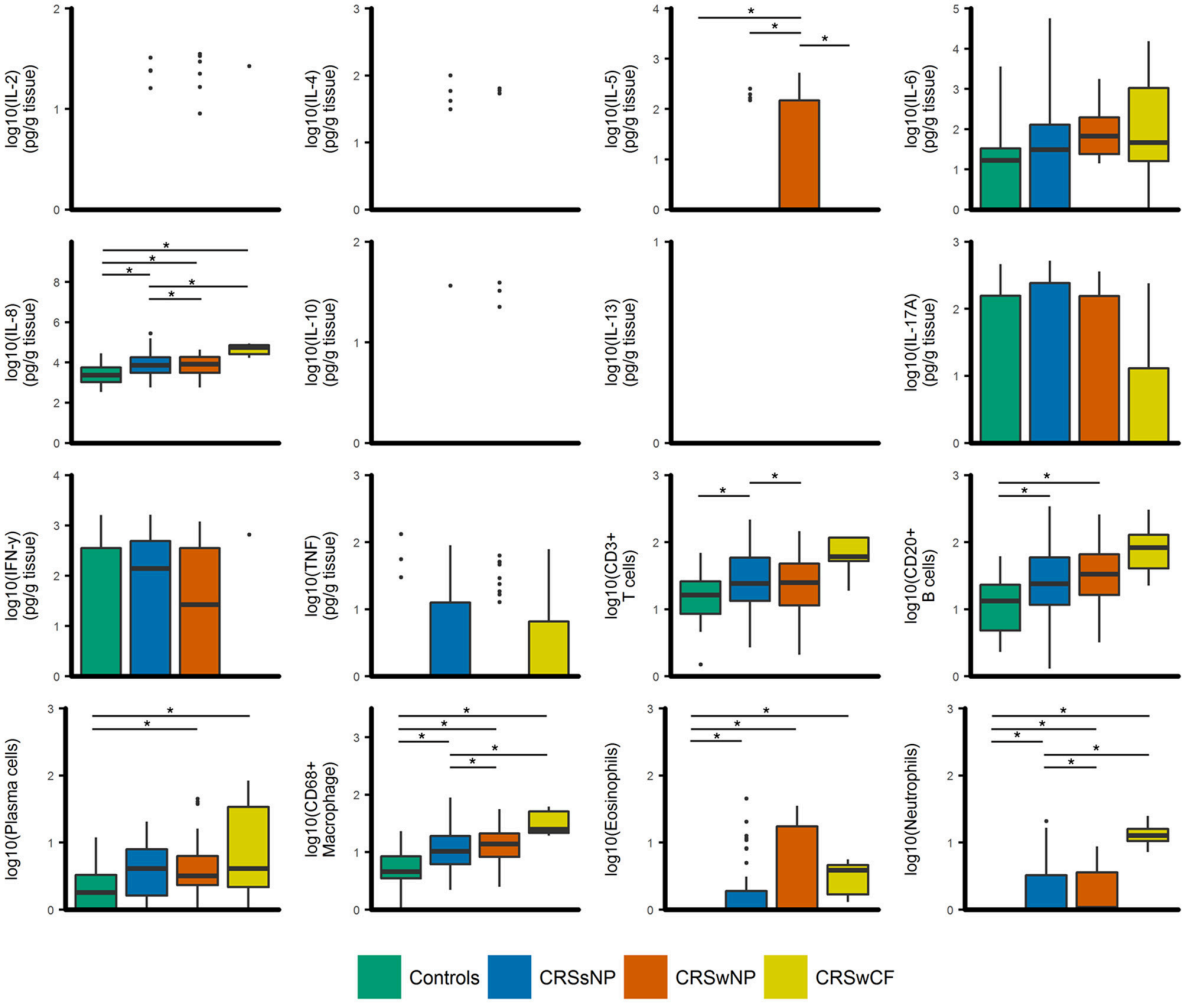
Boxplots of inflammatory variables grouped by CRS phenotypic subtypes [controls (*n* = 17), CRSsNP (*n* = 46), CRSwNP (*n* = 40), CRSwCF (*n* = 7)]. All data are log-transformed for visualization. Values for cell counts are log-transformations of cell numbers per high-powered field. Box center lines represent median values, whiskers outline values up to 1.5 × the interquartile range (IQR), and remaining outlier values are presented as dots. For variables where boxes and whiskers are absent, all values in the range of the box and whisker [IQR + whiskers up to 1.5 × (IQR)] were equal to zero. ^*^represents significant differences between groups (Dunn's test of multiple comparisons with Bonferroni adjustment for multiple comparisons; α = 0.05).

IL-8, CD68^+^ macrophages, eosinophils, neutrophils, and plasma cells were significantly elevated in all CRS groups compared to controls (*p*-values < 0.04) [with the exception of plasma cells in CRSsNP subjects (*p* = 0.053)] (Figure 1). IL-8, CD68^+^ macrophages and neutrophils were also significantly elevated in CRSwNP and CRS with cystic fibrosis (CRSwCF) subjects compared to CRSsNP subjects (*p-*values < 0.05). CRSwNP subjects had significantly higher IL-5 than all other groups (*p*-values < 0.03), more CD20^+^ B cells compared to controls (*p* = 0.011), and more CD3^+^ T cells than CRSsNP subjects (*p* = 0.043). CRSsNP subjects had significantly higher CD20^+^ B cells and CD3^+^ T cells than controls (*p*-values < 0.005). Significant pairwise comparisons are given in a supplementary table available online (Supplementary Table 1).

Principal components analysis (PCA) of the inflammatory variables identified six principal components that cumulatively explained 74% of the variance in the data. Principal components comprised the following: PC1. IL-17A, IFN-γ, IL-4, and TNF; PC2. CD3^+^ T cells, CD20^+^ B cells, and CD68^+^ macrophages; PC3. IL-5 and eosinophils; PC4. IL-6, IL-8, and neutrophils; PC5. IL-10 and IL-2; and PC6. plasma cells. Partitioning of variables in hierarchical clustering was broadly similar (Supplementary Figure 2).

### CRS endotypes

**Figure 2:**
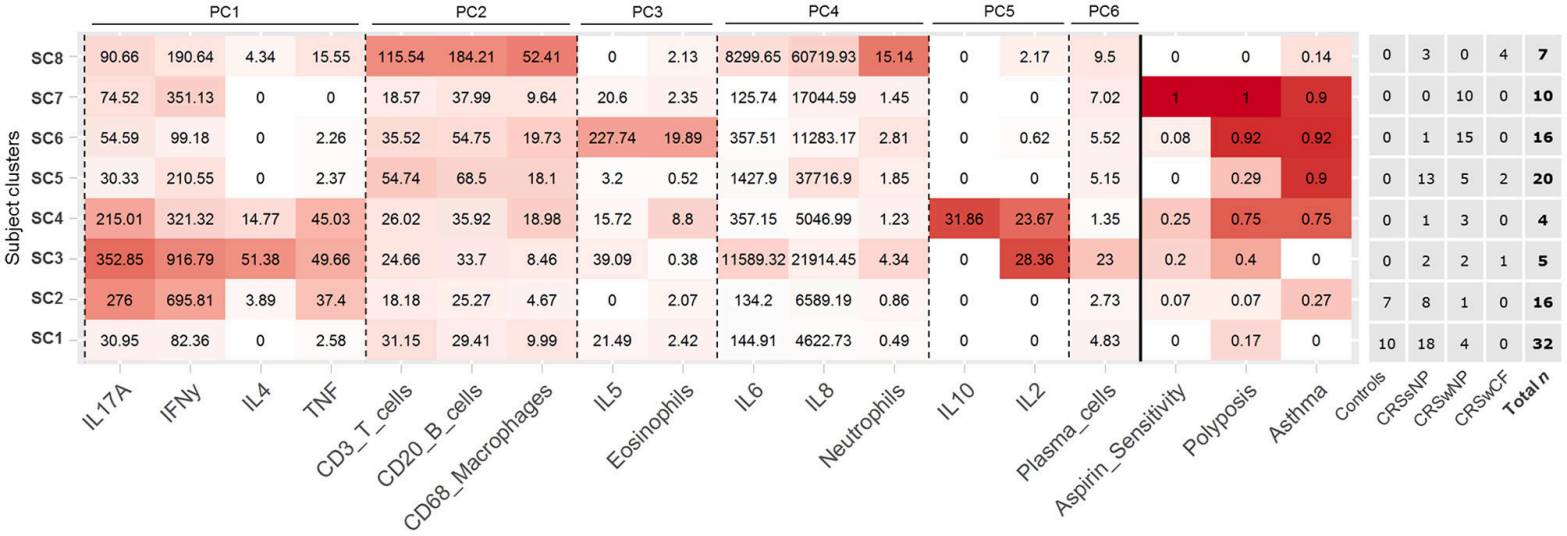
Heat map depicting patterns of inflammatory variables and comorbidities across the eight identified subject clusters [SC1 (*n* = 32), SC2 (*n* = 16), SC3 (*n* = 5), SC4 (*n* = 4), SC5 (*n* = 20), SC6 (*n* = 16), SC7 (*n* = 10), SC8 (*n* = 7)]. All variables in the heat map were included in the subject clustering analysis. Partitioning of subjects across clinical phenotypes (based on polyposis and cystic fibrosis), and total *n* for each cluster are given to the right. Variables for each cluster are color coded based on the mean value of the cluster for each variable, with intensity based on a scale of zero to maximum recorded value for each variable. Variables are arranged by principal components analysis results (PC1-6). Subject clusters are ordered based on general similarities.

Partitioning of subjects into eight subject clusters (SC1-8) gave the highest support (bootstrap means range: 0.63–0.92), and this was chosen for subsequent analyses. Control subjects were divided across SC1 and SC2 together with a number of CRS subjects (Figure 2). In general, subject clusters and traditional CRS phenotypes (CRSsNP, CRSwNP, and CRSwCF) were broadly congruent, but did not align directly, with subjects in all groups spread across a range of subject clusters.

#### Demographics and inflammatory profiles

**Figure 3:**
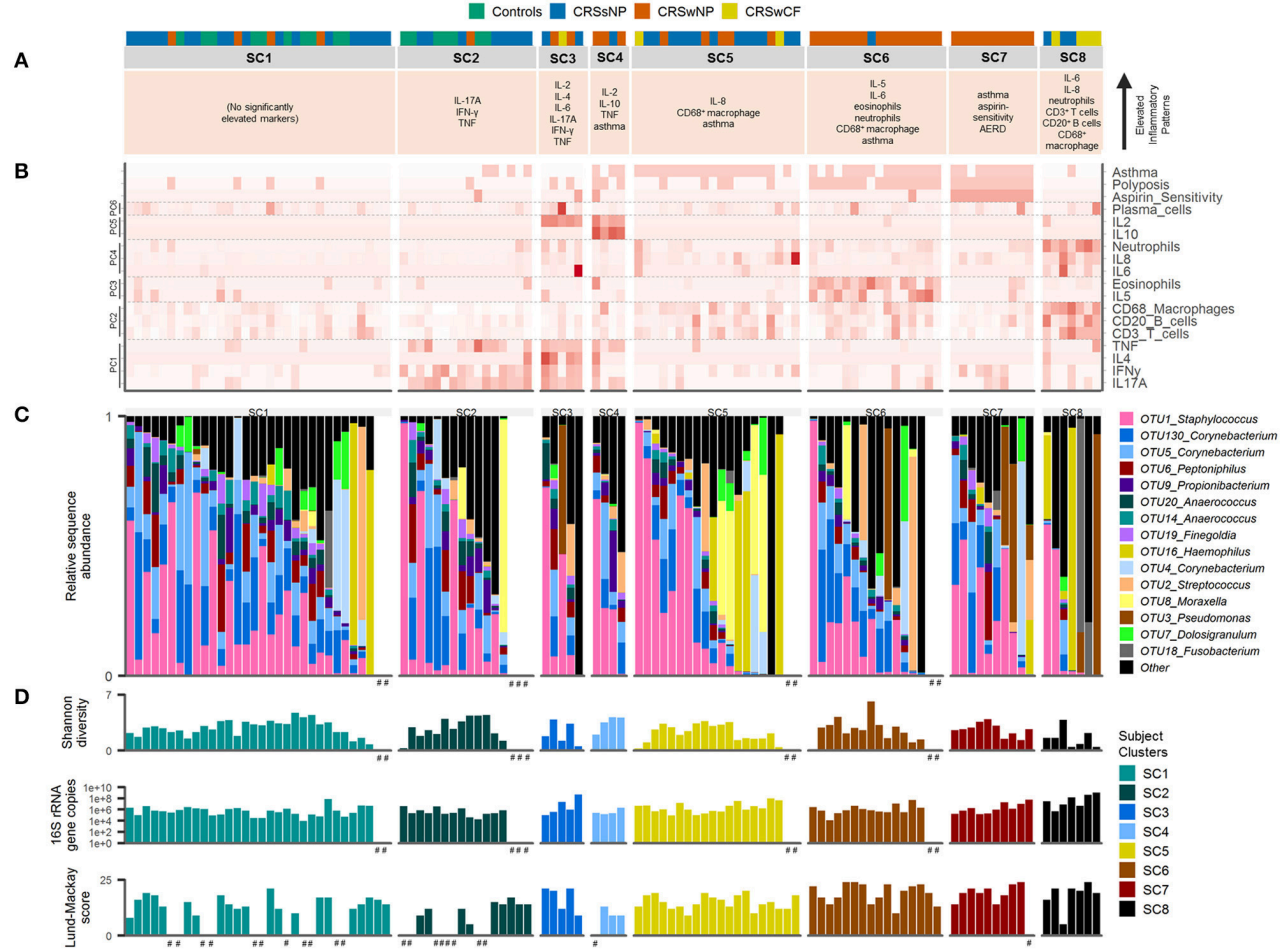
Inflammatory markers and bacterial communities across subject clusters [SC1 (*n* = 32), SC2 (*n* = 16), SC3 (*n* = 5), SC4 (*n* = 4), SC5 (*n* = 20), SC6 (*n* = 16), SC7 (*n* = 10), SC8 (*n* = 7)]. **(A)** Characteristic inflammatory patterns reflecting significantly elevated variables (α = 0.05) compared with at least one other of the eight subject clusters. Color coding above denotes subject phenotype based on polyposis and cystic fibrosis [controls (*n* = 17), CRSsNP (*n* = 46), CRSwNP (*n* = 40), CRSwCF (*n* = 7)]. **(B)** Heat map depicting subject profiles for each of the variables included in the subject clustering. Variables are arranged by principal components analysis results (PC1-6). **(C)** Taxonomic summary plot of bacterial community assemblages for each subject. **(D)** Bacterial community diversity (Shannon diversity index) and total bacterial load (shown on a log scale) based on data from Hoggard et al. (13), and Lund-Mackay clinical severity scores for each patient. Subjects within each cluster are arranged in descending order of the sum of the relative abundances of the eight “healthy-associated” bacterial OTUs discussed in this study. ^#^no data available.

Each subject cluster was characterized by distinct profiles of inflammatory variables (Figure 2). Patterns of inflammatory variables, bacterial community data, and characteristic elevated inflammatory patterns for subject clusters (based on significant differences with at least one other subject cluster) are presented in Figure 3. Differences were observed between some subject clusters for the variables age, Lund-Mackay score, concomitant asthma and aspirin sensitivity. There were no significant pairwise differences for any subject clusters in terms of antibiotics or corticosteroids usage in the 4 weeks prior to surgery (Supplementary Tables 2, 3).

#### Bacterial communities

**Figure 4:**
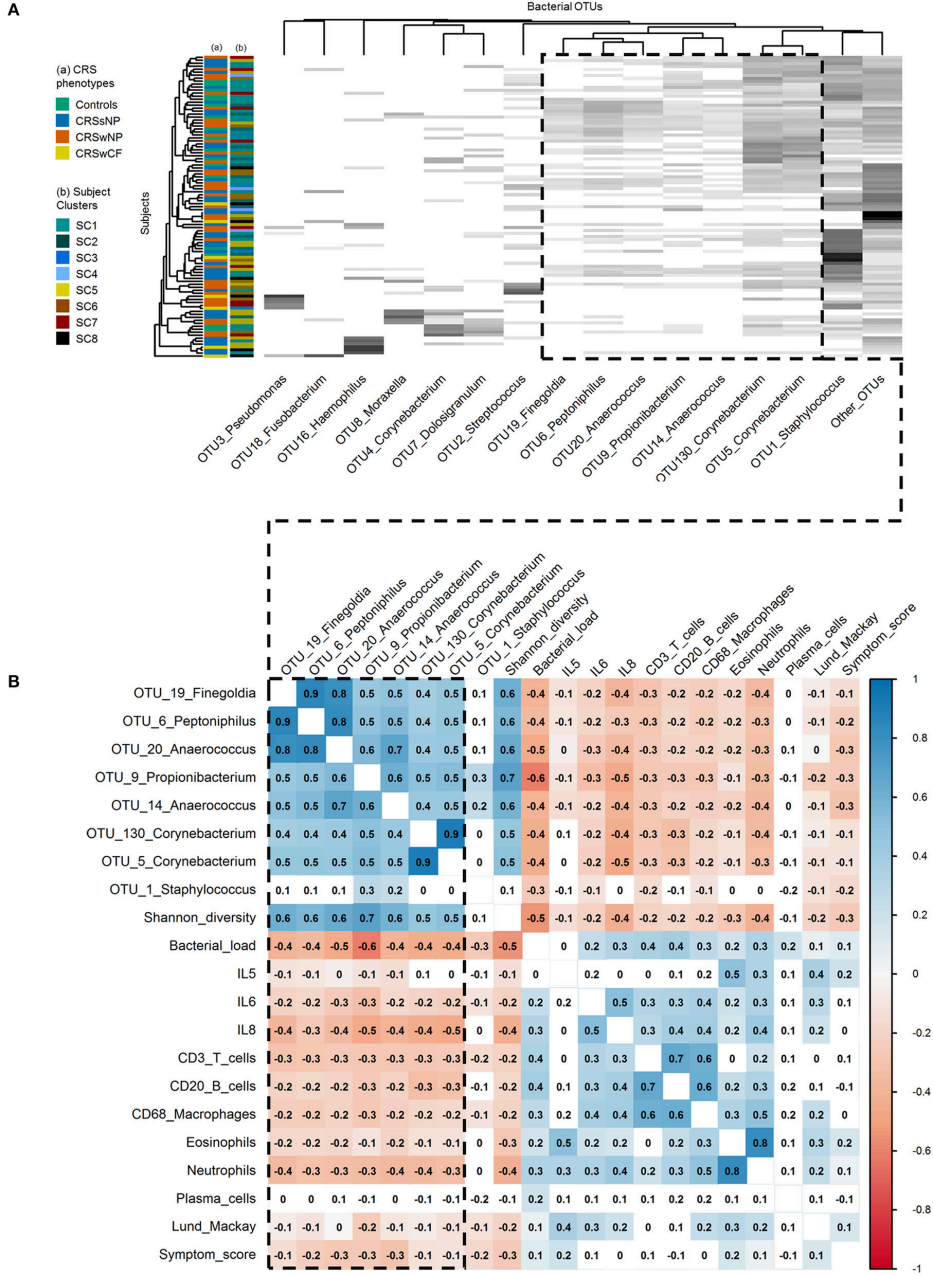
Associations within and between bacterial communities and key inflammatory variables. **(A)** Heat map of the top 15 bacterial OTUs by subject. Ordering of subjects (rows) and bacterial OTUs (columns) were determined using average linkage hierarchical clustering of bacterial community similarity (based on Bray-Curtis dissimilarity), and co-occurrence patterns of bacterial OTUs, respectively: the clustering of the dendrogram branches indicates subjects or bacterial OTUs that are most similar to each other. Heat map values are shaded from white to black according to increasing relative sequence abundance of bacterial OTUs within each subject (white = 0%, black = 100%). Subject color codes are for **(A)** the phenotypic divisions of Controls (*n* = 17), CRSsNP (*n* = 46), CRSwNP (*n* = 40), CRSwCF (*n* = 7), and **(B)** the eight subject clusters identified in this study [SC1 (*n* = 32), SC2 (*n* = 16), SC3 (*n* = 5), SC4 (*n* = 4), SC5 (*n* = 20), SC6 (*n* = 16), SC7 (*n* = 10), SC8 (*n* = 7)]. (**B**) Heat map of Spearman correlations between key bacterial and immunological variables. Color-coding represents significant positive (blue; scale 0 to 1) and negative (red; scale 0 to −1) correlations. Significance testing was based on upper tail probabilities, using the R function cor.test (α = 0.05). Non-significant pairwise associations are uncolored. The dashed box in both denotes the group of eight “health-associated” bacterial OTUs discussed in this study.

Among the 15 most abundant bacterial operational taxonomic units (OTUs) identified, there were few significant differences between subject clusters (Supplementary Table 3). Mean relative abundances of OTUs of *Haemophilus, Pseudomonas*, and *Streptococcus* were high in several subject clusters (Figure 3). However, most of these differences were not significant in subsequent pairwise comparisons testing (with the exception of OTU2_*Streptococcus*, which was significantly elevated in SC4 and SC7 compared with SC8, Supplementary Table 3). Calculation of the predictive probabilities of each OTU occurring in any given subject cluster (presence/absence) further indicated distinctions between the clusters for some OTUs. The presence/absence of OTU16_*Haemophilus* and OTU3_*Pseudomonas* were significantly different across clusters in analyses of deviance (*p*-values < 0.04), and were more characteristic of SC7 and SC8 (probabilities > 0.5 for these two subject clusters). The presence of several OTUs, including representatives of *Staphylococcus, Corynebacterium, Peptoniphilus, Streptococcus, Propionibacterium, Anaerococcus*, and *Finegoldia* was more characteristic of SC1, 2, 4, 5, 6, and 7 (Supplementary Table 4). Grouping subjects by the eight subject clusters in non-metric multidimensional scaling visualization (Supplementary Figure 4) or hierarchical clustering of subjects' bacterial communities (Figure 4A) did not offer additional clarity beyond traditional phenotyping (based on polyposis) or when subjects are grouped by asthma incidence (13).

Spearman correlation and hierarchical clustering analyses across the entire cohort identified an important group of eight bacterial OTUs, with significant positive correlations between OTU19_*Finegoldia*, OTU6_*Peptoniphilus*, OTU20_*Anaerococcus*, OTU14_*Anaerococcus*, OTU9_*Propionibacterium*, OTU130_*Corynebacterium*, and OTU5_*Corynebacterium* (Spearman rank correlation coefficients [rho] of significant pairwise associations ranging from 0.4 to 0.9), and to a lesser extent OTU1_*Staphylococcus* (Figure 4B). With the exception of OTU1_*Staphylococcus*, each of these OTUs was also significantly positively correlated with bacterial community alpha diversity (Shannon diversity index: 0.5 < rho < 0.7), and significantly negatively correlated with bacterial load (−0.4 < rho < −0.6). Alpha diversity was also significantly negatively correlated with bacterial load (rho = −0.5). This was similarly shown in hierarchical clustering of OTUs, where these OTUs clustered together reflecting co-occurrence patterns (Figure 4A). In hierarchical clustering of subjects based on bacterial community similarity, these taxa regularly occur together in subjects in the upper portion of the heat map (Figure 4A), along with fewer instances of the other bacterial OTUs assessed. Notably, a depletion of some of the diversity of these eight OTUs (as seen in subjects lower down the heat map) was associated with dominance by one of the other taxa, including several putative pathogens such as OTUs of *Staphylococcus, Streptococcus, Pseudomonas, Moraxella, Haemophilus*, and *Fusobacterium*.

#### Associations between bacterial OTUs and inflammatory markers

A depletion of the aforementioned bacterial OTUs, reduced bacterial community diversity, and elevated bacterial load were each associated with several markers of inflammation, and with higher clinical severity scores. IL-6, IL-8, CD3^+^ T cells, CD20^+^ B cells, CD68^+^ macrophages, eosinophils, neutrophils, Lund-Mackay scores, and symptom scores [and to a lesser extent, IL-5 (−0.1 < rho < 0)] were each significantly negatively correlated with the OTUs noted above (with the exception of OTU1_*Staphylococcus*) as well as overall bacterial community diversity (−0.5 < rho < −0.2), and significantly positively correlated with overall bacterial load (0.1 < rho < 0.4) (Figure 4B).

## Discussion

**Figure 5:**
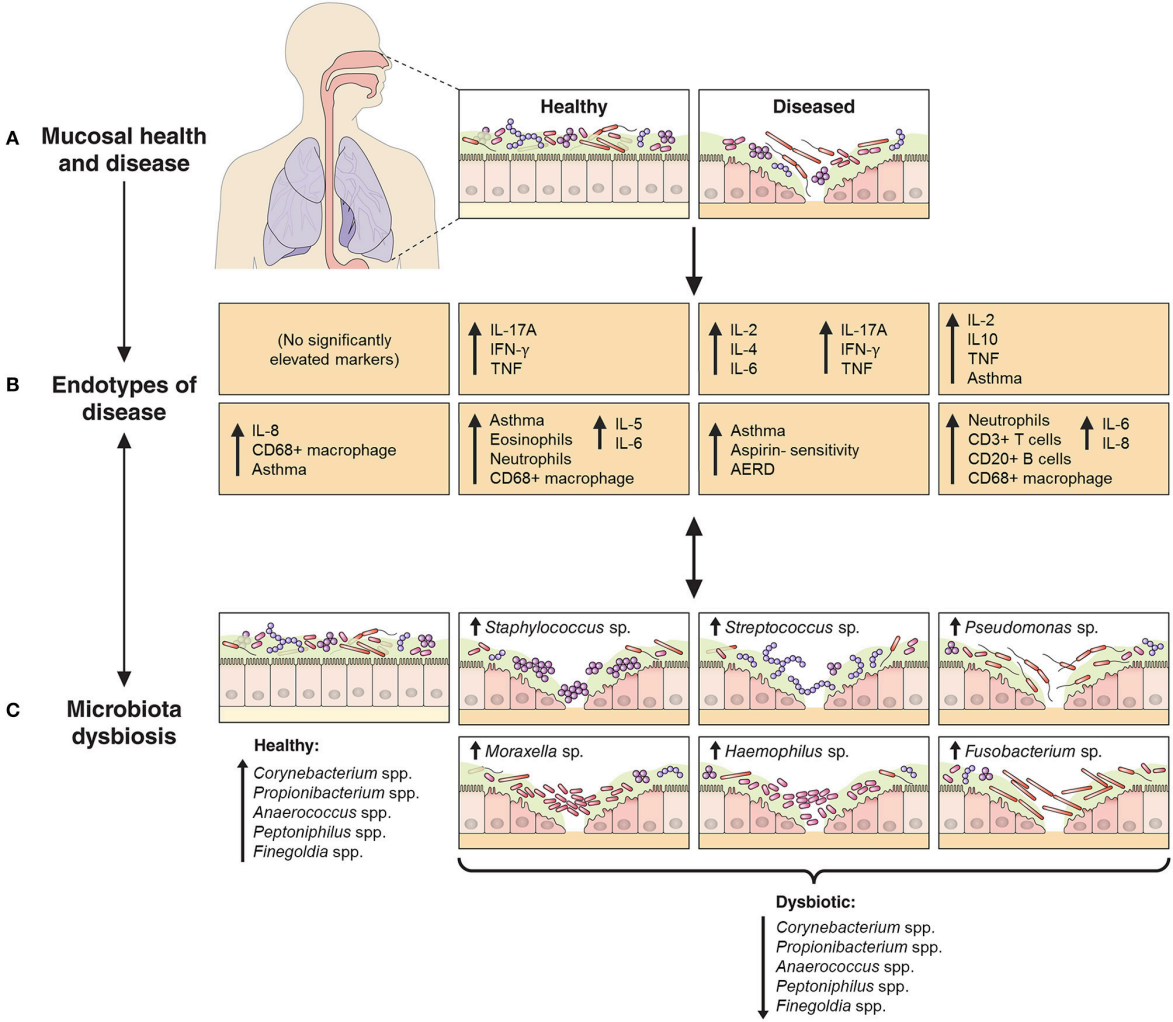
Schematic representation of the relationship between endotypes as sub-types of chronic inflammatory respiratory disease and associated patterns in microbial community dysbiosis (instability and disturbance). The upper panels **(A,B)** depict the shift from a binary (health vs. disease) approach to define and treat chronic inflammatory mucosal diseases to identifying multiple distinct endotypes (disease variants) on the basis of inflammatory biomarkers. The extent to which aberrant patterns in microbial communities (**C**, bottom panels) associate with or drive the underlying inflammatory heterogeneity is of increasing interest. As an example to further provide context, findings from this study of chronic rhinosinusitis subjects have been incorporated into the model: endotypes are represented by the characteristic elevated inflammatory patterns of the eight subject clusters identified in this study, including inflammatory signaling profiles and incidence of comorbidities such as asthma and polyposis. Bacterial community dysbiosis, here characterized by a loss of several “health-associated” taxa (including *Staphylococcus* spp., *Corynebacterium* spp., *Propionibacterium* spp., *Anaerococcus* spp., *Peptoniphilus* spp., and *Finegoldia* spp.) together with dominance by one of several putative pathogens (including particular members of the genera *Staphylococcus, Streptococcus, Pseudomonas, Moraxella, Haemophilus*, and *Fusobacterium*), was associated with elevated inflammatory markers and clinical severity scores. A direct relationship between particular dysbiotic bacterial community types and specific endotypes of CRS remains unclear.

Recent studies have begun to dissect the heterogeneity of inflammatory processes underlying CRS (10–12, 24). These studies have identified the need for more accurate demarcation of CRS variants across a multidimensional array of inflammatory axes. We assessed mucosal inflammatory profiles in 93 CRS subjects and 17 non-inflammatory controls with the aim of resolving CRS subjects on the basis of putative endotypes. In addition, we investigated bacterial associations with specific inflammatory markers, and the extent to which endotypes might better clarify observed patterns compared to previous efforts with standard phenotypes alone (13) (Figure 5).

### Inflammatory patterns in CRS

#### Inflammatory patterns of CRS phenotypes

CRSsNP and CRSwNP were previously described as generally characterized by T_h_1/neutrophilic and T_h_2/eosinophilic inflammation, respectively (24–26). However, reports of the proportion of eosinophilia associated with nasal polyposis in European cohorts have ranged from 54 to 90% (8, 27), and in Asian populations as low as 7.5% (27). There is increasing recognition that standard phenotypic divisions of CRS and the binary model of T_h_1 vs. T_h_2 inflammation are insufficient to describe the inflammatory heterogeneity in CRS (6–11). This was reflected in this study: inflammatory patterns were highly variable both between and within standard phenotypic variants of CRS. While there were indications of significantly elevated inflammatory markers in some phenotypes compared to others (including IL-5, IL-8, CD3^+^ T cells, CD68^+^ macrophages, and neutrophils), median values for several, including IL-5, eosinophils, and neutrophils, were zero in many cases. This highlights that, while there were increased incidences of these types of inflammation, they were absent in more than half of the subjects in these phenotypic groups. Thus, “increased incidence” of neutrophilia or eosinophilia (or elevated IL-5, etc.) is a more accurate representation of these phenotypic groups than the common description that each is “characterized” by T_h_1/neutrophilic or T_h_2/eosinophilic inflammation.

#### Clinical implications

These observations may explain some of the difficulties encountered in treating CRS: targeted treatment may be difficult when inflammatory processes underlying the disease are heterogeneous. While there has been increasing interest in targeted treatments such as monoclonal antibodies in chronic inflammatory conditions such as CRS (28–31), treatments aimed narrowly at specific mechanisms of inflammation may have limited efficacy or applicability across the group as a whole, where the targeted mechanism may not be relevant in sizeable proportions of subjects, or where mixed inflammatory processes might be at play in parallel within individual subjects. In contrast, the relative efficacy of corticosteroids may derive from their action of dampening inflammatory processes more broadly. For example, previous studies have identified CRSwNP as generally having elevated IL-5 (24, 26). In this study, mean IL-5 was similarly elevated for the group overall, however, the median value was zero. Thus, anti-IL-5 monoclonal antibody treatment (such as Mepolizumab) might be expected to be effective or appropriate in fewer than half of CRSwNP subjects. Monoclonal antibody trials and treatments directed by first characterizing individual patients' inflammatory patterns within the affected mucosa may better determine appropriate personalized treatment options.

#### Key inflammatory variables in CRS

IL-8, CD68^+^ macrophages, eosinophils, neutrophils and plasma cells were significantly elevated in all three CRS groups (CRSsNP, CRSwNP, CRSwCF), and IL-5 was significantly elevated in CRSwNP. IL-6 and IFN-γ (together with IL-5 and IL-8) were also important variables in identified subject clusters, and in Spearman correlation analysis of key bacterial OTUs, inflammatory variables, and clinical severity scores. Thus, these data provide further evidence for IL-5, IL-6, IL-8, IFN-γ, TNF, and comorbidities including asthma, polyposis, and aspirin sensitivity, in addition to the previously identified IL-13, albumin, myeloperoxidase, eosinophilic cationic protein, IgE, and SE-IgE (10, 11), as important variables in efforts to demarcate distinct endotypes of CRS.

#### Endotypes of CRS

Eight subject clusters (SC1-8) were identified in this study. Overall, subject clusters were roughly divided into either low or high rates of asthma and/or polyposis incidence. This is in keeping with Tomassen and colleagues, who found that endotypes largely adhered to clinical phenotypes based on polyposis (10). In contrast to their findings, however, a clear differentiation of subject clusters into 3 subgroups on the basis of IL-5 levels (no IL-5, moderate IL-5 in all subjects, and high IL-5 in all subjects) was not seen in this cohort. Why this pattern was not reproduced in this study is unclear, but may reflect methodological differences between the studies, or genuine underlying differences between the study cohorts. Differences in CRS inflammatory profile types have been observed previously between different populations globally, including an increased association between polyposis and eosinophilia and IL-5 in European populations compared to patients studied in China (27). Patterns of association for IL-5 may similarly be less prominent in this New Zealand based cohort.

Notably, controls did not fall into their own subject clusters, but instead clustered together with a number of CRSsNP subjects and a handful of CRSwNP subjects. This further highlights the complicated nature of the inflammatory patterns underlying CRS, and in particular CRSsNP, and the need to include a broad range of variables when attempting to delineate CRS endotypes.

Ageing has an important influence on the immune system through the process of gradual immunosenescence, including dysregulation and reduced efficiency in managing chronic inflammation (32, 33). It is unclear whether differences in age may influence the progression and types of inflammation underlying CRS, and whether it may underpin some of the observed distinctions in inflammatory profiles across the subject clusters in this study. Significant differences in age were observed between some subject clusters in this study. However, this was in particular due to SC8, which included several CRSwCF patients. As CRSwCF also tends to be a younger patient group overall (due to the underlying congenital condition driving earlier onset of CRS), this likely confounds the observed differences in age between the subject clusters here.

To the extent that only a handful of potential inflammatory biomarkers in CRS have been examined, the results so far cannot be taken to categorically define specific endotypes of CRS, but rather provide early indications of where the inflammatory lines might be drawn and provide proof of principle. It is important to note that numbers for some subject clusters in this study were small: SC3, SC4, SC7, and SC8 only contained 5, 4, 10, and 7 subjects each. Additionally, suitable non-CRS control subjects for whom mucosal biopsies can be collected are comparably rare and difficult to recruit in large numbers, and included controls, while exhibiting no signs of CRS, are nonetheless undergoing endoscopic sinus surgery for other reasons, and may not fully represent “healthy” subjects. Observed similarities and deviations from this group when taken as a reference baseline of “normal” healthy mucosal inflammatory profiles should be viewed in light of these caveats. Furthermore, cross-sectional sampling may obscure whether some of the observed differences in subject clusters represent a temporal continuum of specific endotypes as they develop over time. Given the observed inflammatory complexity, together with other potential sources of influence in determining disease subtypes such as genetic (34) and life history factors (35, 36), many more subjects measured over more variables (or more refined variables) and at multiple time points will be necessary to clearly delineate between true endotypes of CRS and to subsequently draw robust conclusions from comparisons.

#### Implications for hypotheses of CRS

Numerous cases of mixed inflammatory patterns were seen within individual subjects, a phenomenon which has also been identified previously (9). This observation may lend support to a model involving loss of inflammatory regulation underlying the diverse variants of CRS, rather than a specific pro-inflammatory mechanism. Evidence suggestive of a dysfunction of T cell regulation in CRSwNP has been observed previously (25, 37, 38).

A loss of mucosal integrity or predisposition toward a defective immune barrier may also be central to the pathogenesis of CRS (39). Decreased expression of tight junction proteins has been identified in CRSwNP (40, 41), and specific inflammatory molecules that are elevated in CRS have been linked to mucosal epithelial disruption. Oncostatin M, a member of the IL-6 family of cytokines, can drive increased mucosal permeability and mislocalization of tight junction proteins, together with eosinophil infiltration and the production of IL-4, IL-5, and IL-13 (42). Similar disruption has also been seen with IL-4, IFN-γ, IL-17, IL-22, and IL-26 (41, 43). In this study, IL-4, IL-5, and IL-17 were markedly elevated in certain subjects, with significantly elevated IL-4 in SC3, IL-17 in SC2 and SC3, and IL-5 in all CRSwNP subjects. Importantly, compromised mucosa would allow infiltration of diverse microbial and/or antigenic agents into the sub-mucosa, and may drive the mixed inflammatory patterns observed in CRS subjects here and elsewhere.

### Bacterial associations with inflammation in CRS

#### Bacterial associations with CRS endotypes

Attempts to identify specific, putatively pathogenic bacterial associations with phenotypic variants of CRS have remained largely unsuccessful (39, 44). We hypothesized that aberrant bacterial taxa or community types may more closely associate with inflammatory endotypic variants than with phenotypic variants of CRS. However, reanalysis of previously published bacterial community data for this cohort identified few positive associations with inflammatory variables or endotypes. Several taxa of interest, including OTUs of *Haemophilus, Streptococcus*, and *Pseudomonas*, did have high mean relative abundances in a number of subject clusters. However, these differences were not significantly different in pairwise comparisons testing. Whether this is a factor of too few subjects in some groups, or simply reflects a limited relation overall between inflammatory patterns and particular bacterial taxa, remains unclear. Notably, such associations between bacterial community types and particular inflammatory markers were observed in a recent study by Cope et al. (12), suggesting an even more specific link between the type of microbial dysbiosis and the resultant inflammatory endotype of CRS. Together with the data presented in this study, these findings provide increasing support for an association between microbial community dysbiosis and inflammation generally, as well as an indication of links between specific bacterial community types and possible endotypes of CRS.

#### Bacterial and inflammatory correlations

A depletion of a group of bacterial taxa, including OTUs of *Anaerococcus, Propionibacterium, Corynebacterium, Staphylococcus, Peptoniphilus*, and *Finegoldia*, together with lower bacterial diversity overall and increased bacterial load, broadly associated with increased inflammatory markers and higher clinical severity scores. These taxa were previously identified within this cohort as being more typical of health-associated bacterial communities (with aberrant community types tending to occur more often in CRS) (13, 45). These data provide further evidence for the potential role of microbial dysbiosis (instability and disturbance) in CRS progression or exacerbation, where community shifts away from a normal healthy community may drive mixed inflammatory responses [such as the range of toll-like receptor (TLR) activities identified previously in CRS (17, 46, 47)], in contrast to singular pathogenic activities (such as superantigens) driving very specific inflammatory responses. Overall, aberrant microbial communities, coupled with increased mucosal damage and permeability in CRS, might further drive the heterogeneous inflammatory patterns observed.

A range of key inflammatory markers were assessed in this study, providing further evidence of the inflammatory heterogeneity underlying CRS. However, the overall picture remains unclear. More comprehensive assessment of the multitude of inflammatory processes within the mucosa beyond those assessed both here and previously may better distinguish between true endotypes of CRS, as well as identify specific associations with polyposis or asthma and clarify genuine microbial associations across the spectrum of CRS.

## Summary and concluding remarks

This study contributes to ongoing efforts to further refine distinct endotypes of CRS on the basis of underlying inflammatory processes. Inflammatory patterns were highly variable within standard phenotypic variants of CRS, and mixed inflammatory profiles were often observed. In contrast, eight subject clusters, which included both control and CRS subjects, were identified with distinct patterns of inflammation and associated comorbidities. In addition to previously identified variables, the data provide further evidence for the importance of IL-2, IL-5, IL-6, IL-8, IL-10, IFN-γ, TNF, asthma, polyposis, and aspirin sensitivity, in efforts to demarcate distinct endotypes of CRS. Finally, reanalysis of previously published bacterial data for this cohort identified an important association between the depletion of a “health-associated” group of bacterial taxa, reduced bacterial diversity and increased bacterial load overall, and markers of inflammation and clinical severity. Taken together with other recent work on inflammatory endotypes and microbial community associations in CRS (10, 12), these data further establish the importance of defining distinct endotypes of CRS, as well as provide an early indication of a connection between the microbiota and the inflammatory heterogeneity of CRS.

## Author contributions

MH contributed to study design, the acquisition of data, data analyses, and writing of the manuscript. SW-T contributed to histological processing and the acquisition of inflammatory cell data. MZ and RD contributed to subject recruitment and sampling. KC contributed to statistical analyses. FR contributed to the acquisition of inflammatory signaling data. BW contributed to data analyses. MT, RD, and KB contributed to study design, and MT, RD, KB, and FR provided laboratory space and materials. All authors contributed to editing of the manuscript.

### Conflict of interest statement

The authors declare that the research was conducted in the absence of any commercial or financial relationships that could be construed as a potential conflict of interest.
