# Supplementary material for: Inflammatory Endotypes and Microbial Associations in Chronic Rhinosinusitis
Source: Front Immunol. 2018 Sep 19;9:2065. doi: 10.3389/fimmu.2018.02065 (PMC6157407; doi:10.3389/fimmu.2018.02065)
Supplement: Supplementary file 1 [file Presentation_1.PDF]

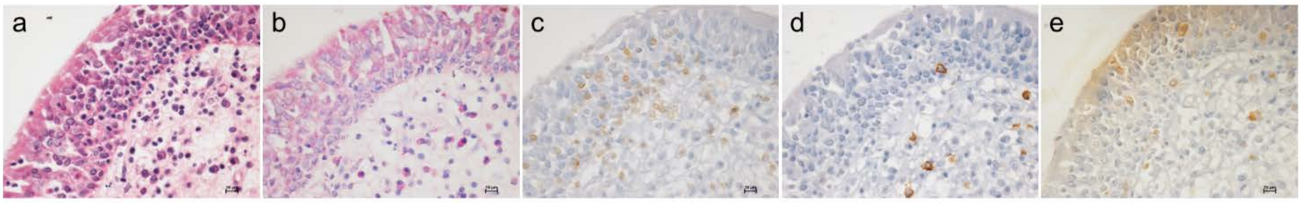

Supplementary Figure 1. Representative sections for each of the six inflammatory cells assessed: **a.** eosinophils and neutrophils (H&E); **b.** plasma cells (methyl green-pyronin staining); **c.** $CD3^{+}$ T cells; **d.** $CD20^{+}$ B cells; **e.** $CD68^{+}$ macrophages.

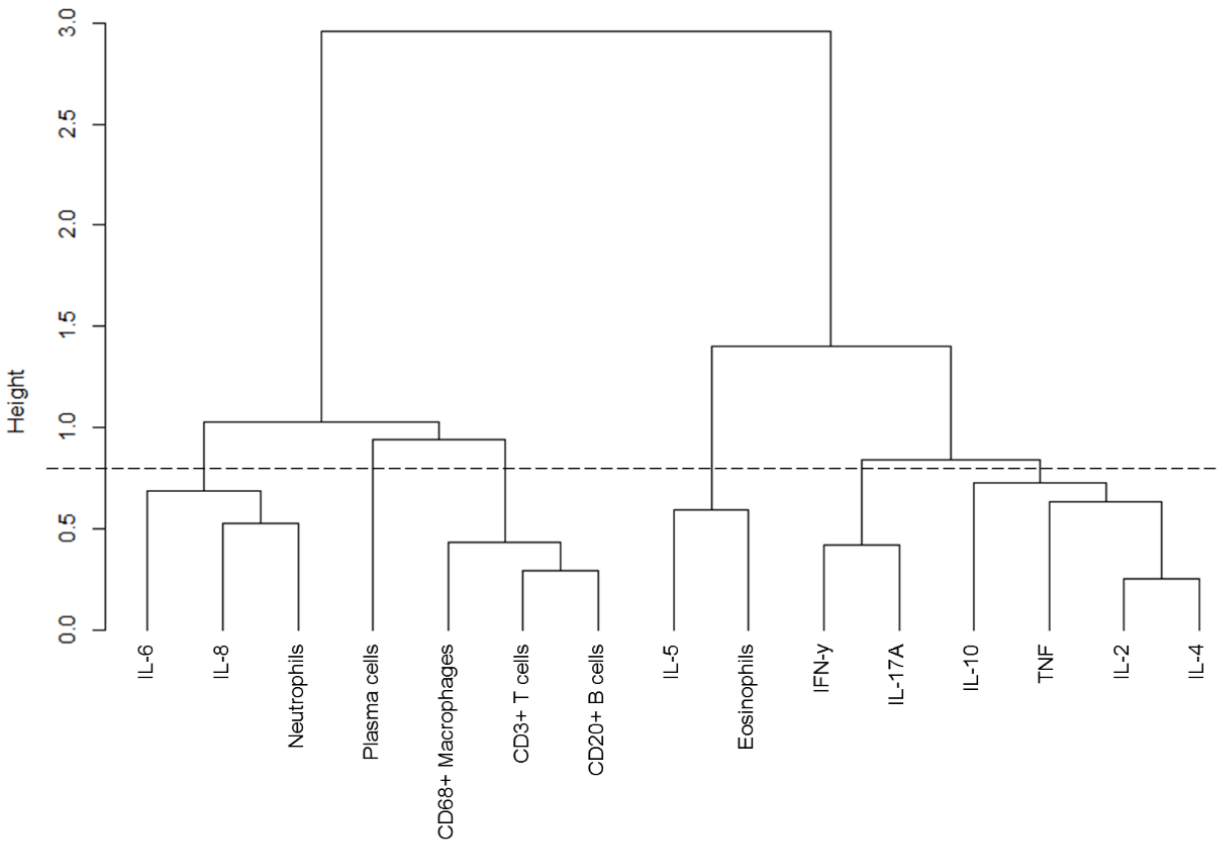

Supplementary Figure 2. Hierarchical Clustering of inflammatory variables. The dashed line represents the cutoff threshold partitioning the variables into six clusters.

(**A**)

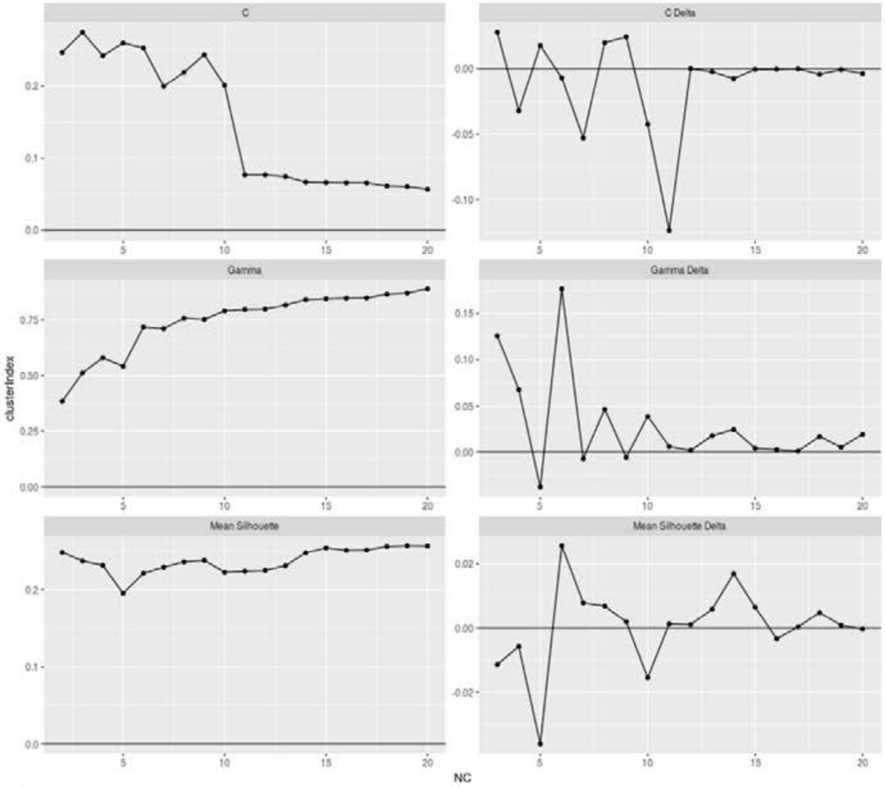

(**B**)

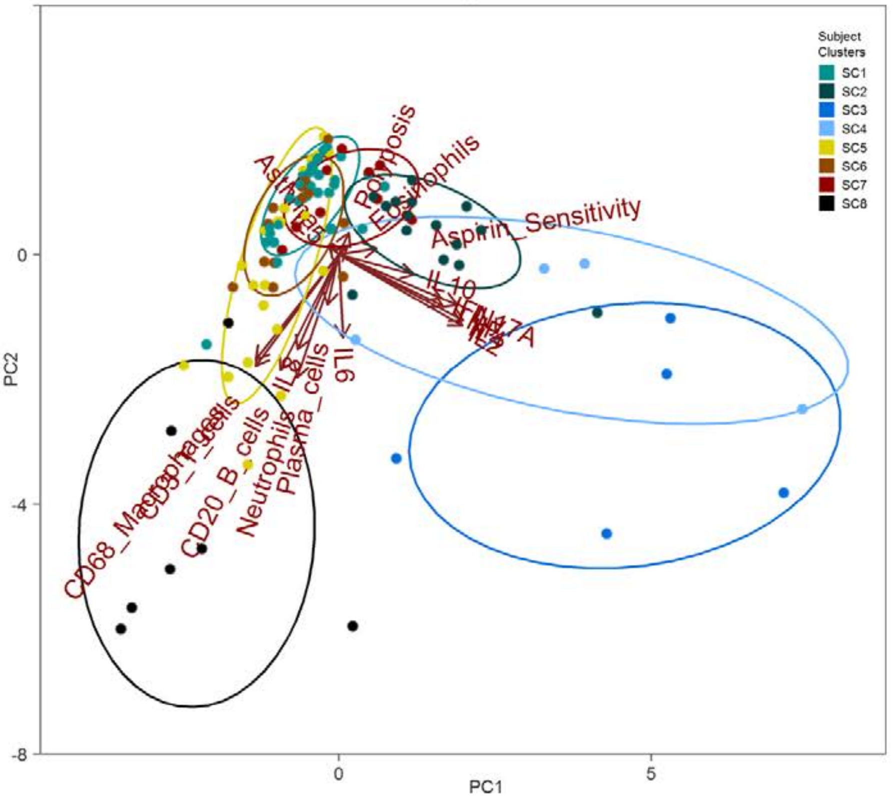

Supplementary Figure 3. Subject Clustering plots. (**A**) Clustering index plots used to inform optimal number of subject clusters. (**B**) Plot of top two principal components, with subjects grouped by eight clusters (SC1 (*n* = 32), SC2 (*n* = 16), SC3 (*n* = 5), SC4 (*n* = 4), SC5 (*n* = 20), SC6 (*n* = 16), SC7 (*n* = 10), SC8 (*n* = 7)), overlain with the variables incorporated into the subject clustering analysis.

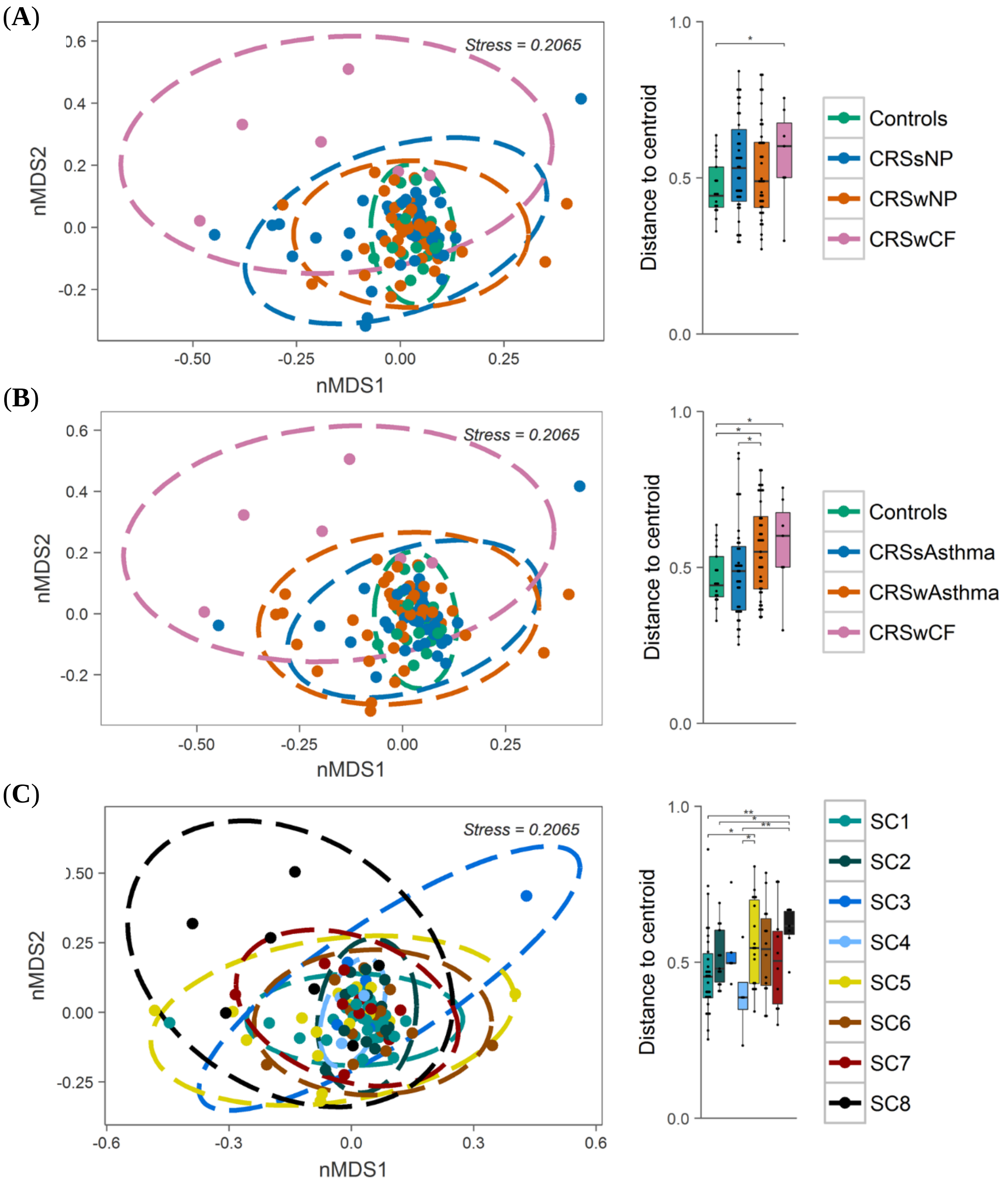

Supplementary Figure 4. Bacterial community differences between subjects. Non-metric multidimensional scaling plots and boxplots of bacterial community differences, with subjects grouped by: (**A**) phenotypes based on concomitant polyposis and cystic fibrosis (Controls (n = 17), CRSsNP (n = 46), CRSwNP (n = 40), CRSwCF (n = 7)); (**B**) phenotypes based on concomitant asthma or cystic fibrosis (Controls (n = 17), CRSsAsthma (n = 40), CRSwAsthma (n = 46), CRSwCF (n = 7)); (**C**) eight subject clusters identified in this study (SC1 (n = 32), SC2 (n = 16), SC3 (n = 5), SC4 (n = 4), SC5 (n = 20), SC6 (n = 16), SC7 (n = 10), SC8 (n = 7)). Bacterial community differences are based on Bray-Curtis dissimilarity. Boxplots represent the distances of subjects from the centroid for each group; significant differences identify groups that are significantly differently dispersed (i.e. differences in inter-subject variability within groups) (Dunn's test of multiple comparisons with Bonferroni adjustment for multiple comparisons; α = 0.05).
