# Supplementary material for: Inflammatory Endotypes and Microbial Associations in Chronic Rhinosinusitis
Source: Front Immunol. 2018 Sep 19;9:2065. doi: 10.3389/fimmu.2018.02065 (PMC6157407; doi:10.3389/fimmu.2018.02065)
Supplement: Supplementary file 2 [file Presentation_2.PDF]

Supplementary Table 1. Pairwise comparisons for significant variables*.

		Nonparametric pairwise comparison (Bonferroni-adjusted *p*-value)					
	Variables†	Controls vs. CRSsNP	Controls vs. CRSwNP	Controls vs. CRSwCF	CRSsNP vs. CRSwNP	CRSsNP vs. CRSwCF	CRSwNP vs. CRSwCF
Subject demographics	Age	**0.0036**	0.6052	0.1506	**0.0244**	0.0805	1.0000
	Asthma	0.0754	**0.0000**	1.0000	**0.0310**	1.0000	0.0544
	Aspirin sensitivity	1.0000	0.4903	1.0000	**0.0026**	1.0000	0.9987
	AERD	1.0000	0.1414	1.0000	**0.0006**	1.0000	1.0000
	Preoperative antibiotics‡	1.0000	1.0000	0.0870	1.0000	0.1637	0.0707
	Revision surgery	-	-	-	1.0000	**0.0067**	**0.0335**
	Lund-Mackay score	-	-	-	**0.0004**	0.1895	0.9658
Inflammatory variables	IL-5	1.0000	1.0000	**0.0007**	1.0000	**0.0280**	**0.0003**
	IL-8	**0.0000**	**0.0117**	**0.0087**	**0.0046**	**0.0073**	1.0000
	$CD3^+$ T cells	**0.0022**	0.0938	0.2263	0.0763	**0.0430**	1.0000
	$CD20^+$ B cells	**0.0018**	0.0793	**0.0109**	0.0754	0.2671	0.9813
	$CD68^+$ macrophages	**0.0000**	**0.0217**	**0.0009**	**0.0051**	**0.0473**	0.5637
	Eosinophils	**0.0007**	**0.0242**	**0.0008**	0.0785	0.4373	0.4704
	Neutrophils	**0.0000**	**0.0139**	**0.0071**	**0.0001**	**0.0002**	1.0000
	Plasma cells	0.0530	**0.0267**	**0.0378**	1.0000	1.0000	1.0000

*Significant p values (α = 0.05) for pairwise comparisons are expressed in bold.

†Categorical variables are tested as binary (yes/no) using Fisher's exact test and continuous variables tested using Dunn's test of multiple comparisons, with Bonferroni adjustment for multiple comparisons

‡Antibiotics in the 4 weeks prior to surgery.

AERD = aspirin-exacerbated respiratory disease; CRS = chronic rhinosinusitis; CRSsNP = CRS without nasal polyps; CRSwNP = CRS with nasal polyps; CRSwCF = CRS with cystic fibrosis

Supplementary Table 2. Subjects' demographics grouped by subject clusters.

Variables[*]	Subject clusters								Unadjusted test *p*-value[‡]
	SC1 (*n* = 32)[†]	SC2 (*n* = 16)[†]	SC3 (*n* = 5)[†]	SC4 (*n* = 4)[†]	SC5 (*n* = 20)[†]	SC6 (*n* = 16)[†]	SC7 (*n* = 10)[†]	SC8 (*n* = 7)[†]	
Age	47 (23 to 80)	53 (19 to 84)	38 (37 to 53)	43 (28 to 63)	46 (18 to 65)	52 (25 to 71)	55 (28 to 68)	28 (20 to 41)	**0.0179**
European	24/32 (75%)	13/16 (81%)	4/4 (100%)	3/3 (100%)	12/20 (60%)	15/16 (94%)	7/10 (70%)	7/7 (100%)	0.1942
Gender	15/32 (47%)	11/16 (69%)	2/5 (40%)	0/3 (0%)	10/20 (50%)	7/16 (44%)	6/10 (60%)	4/7 (57%)	0.5457
Asthma	0/32 (0%)	4/16 (25%)	0/5 (0%)	3/4 (75%)	18/20 (90%)	13/16 (81%)	9/10 (90%)	1/7 (14%)	**<0.0001**
Aspirin Sensitivity	0/32 (100%)	1/16 (6%)	1/5 (20%)	1/4 (25%)	0/20 (0%)	1/16 (6%)	10/10 (100%)	0/7 (0%)	**<0.0001**
AERD	0/32 (100%)	0/16 (0%)	0/5 (0%)	1/4 (25%)	0/20 (0%)	1/16 (6%)	9/10 (90%)	0/7 (0%)	**<0.0001**
Preoperative antibiotics[§]	3/32 (9%)	1/16 (6%)	2/5 (40%)	0/3 (100%)	6/20 (30%)	2/16 (13%)	0/10 (0%)	2/7 (29%)	0.123
Preoperative corticosteroids[§]	1/32 (3%)	1/16 (6%)	0/5 (0%)	0/3 (0%)	6/20 (30%)	2/16 (13%)	0/10 (0%)	3/7 (43%)	**0.0193**
Revision surgery[ll]	6/22 (27%)	2/9 (22%)	3/5 (60%)	1/3 (33%)	10/20 (50%)	9/16 (56%)	5/10 (50%)	6/7 (86%)	0.1244
Total symptom score[ll]	14.50 (2 to 25)	14 (2 to 21)	11 (10 to 22)	14 (11 to 20)	15 (2 to 24)	19 (5 to 25)	18 (6 to 24)	13 (5 to 21)	0.7128
Lund-Mackay score[ll]	14 (8 to 21)	14 (5 to 17)	20 (9 to 21)	9 (9 to 13)	15 (9 to 20)	20 (10 to 24)	19 (14 to 24)	20 (5 to 24)	**0.0003**

[*]Categorical variables are summarized as proportion yes/total (%), except for gender, which is given as proportion female. Continuous variables are summarized as median (range).

[†]Total cohort numbers for each group are given. The differences in total numbers for each variable reflect missing data for some subjects.

[‡]Difference between groups tested using Fisher's exact test for categorical variables and the Kruskal-Wallis test for continuous variables. Significant *p*-values (α = 0.05) are expressed in bold.

[§]Antibiotics and/or corticosteroids in the 4 weeks prior to surgery.

[ll]Revision surgery, symptom score and Lund-Mackay score variables exclude control subjects

AERD = aspirin-exacerbated respiratory disease. SC = Subject Cluster.

Supplementary Table 3. Pairwise comparisons for significant variables* grouped by subject clusters

Nonparametric pairwise comparisons between subject clusters (Bonferroni-adjusted *p*-value)

	Variables†	SC5 vs. SC1	SC5 vs. SC6	SC5 vs. SC2	SC5 vs. SC4	SC5 vs. SC8	SC5 vs. SC3	SC5 vs. SC7	SC1 vs. SC6	SC1 vs. SC2	SC1 vs. SC4	SC1 vs. SC8	SC1 vs. SC3	SC1 vs. SC7	SC6 vs. SC2	SC6 vs. SC4	SC6 vs. SC8	SC6 vs. SC3	SC6 vs. SC7	SC2 vs. SC4	SC2 vs. SC8	SC2 vs. SC3	SC2 vs. SC7	SC4 vs. SC8	SC4 vs. SC3	SC4 vs. SC7	SC8 vs. SC3	SC8 vs. SC7	SC3 vs. SC7
Subject demographics	Age	1.00	1.00	1.00	1.00	0.52	1.00	0.51	1.00	1.00	1.00	**0.02**	1.00	1.00	1.00	1.00	**0.03**	1.00	1.00	1.00	0.06	1.00	1.00	1.00	1.00	1.00	1.00	**0.01**	1.00
	Asthma	**0.00**	1.00	**0.00**	1.00	**0.02**	**0.01**	1.00	**0.00**	0.26	**0.02**	1.00	1.00	**0.00**	0.11	1.00	0.14	0.08	1.00	1.00	1.00	1.00	0.10	1.00	1.00	1.00	1.00	0.10	0.06
	Aspirin sensitivity	1.00	1.00	1.00	1.00	1.00	1.00	**0.00**	1.00	1.00	1.00	1.00	1.00	**0.00**	1.00	1.00	1.00	1.00	**0.00**	1.00	1.00	1.00	**0.00**	1.00	1.00	0.31	1.00	**0.00**	0.10
	AERD	1.00	1.00	1.00	1.00	1.00	1.00	**0.00**	1.00	1.00	1.00	1.00	1.00	**0.00**	1.00	1.00	1.00	1.00	**0.00**	1.00	1.00	1.00	**0.00**	1.00	1.00	1.00	1.00	**0.01**	0.06
	Preoperative steroids‡	0.28	1.00	1.00	1.00	1.00	1.00	1.00	1.00	1.00	1.00	0.39	1.00	1.00	1.00	1.00	1.00	1.00	1.00	1.00	1.00	1.00	1.00	1.00	1.00	1.00	1.00	1.00	1.00
	Lund-Mackay score	1.00	0.11	1.00	1.00	0.73	1.00	0.16	0.05	1.00	1.00	0.49	1.00	0.09	**0.02**	**0.04**	1.00	1.00	1.00	1.00	0.12	1.00	**0.02**	0.10	0.71	**0.03**	1.00	1.00	1.00
Inflammatory variables	IL-2	1.00	1.00	1.00	**0.00**	1.00	**0.00**	1.00	1.00	1.00	**0.00**	1.00	**0.00**	1.00	1.00	**0.00**	1.00	**0.00**	1.00	**0.00**	1.00	**0.00**	1.00	**0.00**	1.00	**0.00**	**0.00**	1.00	**0.00**
	IL-4	1.00	1.00	1.00	0.82	1.00	**0.00**	1.00	1.00	1.00	0.71	1.00	**0.00**	1.00	1.00	0.89	1.00	**0.00**	1.00	1.00	1.00	**0.00**	1.00	1.00	**0.01**	1.00	**0.00**	1.00	**0.00**
	IL-5	1.00	**0.00**	1.00	1.00	1.00	1.00	1.00	**0.00**	1.00	1.00	1.00	1.00	1.00	**0.00**	0.17	**0.00**	0.08	**0.00**	1.00	1.00	1.00	1.00	1.00	1.00	1.00	1.00	1.00	1.00
	IL-6	1.00	1.00	0.11	1.00	1.00	1.00	1.00	0.67	1.00	1.00	0.22	0.12	1.00	**0.04**	1.00	1.00	1.00	1.00	0.73	**0.02**	**0.01**	0.21	1.00	1.00	1.00	1.00	1.00	1.00
	IL-8	**0.01**	1.00	0.12	0.75	0.67	1.00	1.00	1.00	1.00	1.00	**0.00**	0.24	0.21	1.00	1.00	**0.03**	1.00	1.00	1.00	**0.00**	0.61	0.80	**0.03**	1.00	1.00	1.00	0.65	1.00
	IL-10	1.00	1.00	1.00	**0.00**	1.00	1.00	1.00	1.00	1.00	**0.00**	1.00	1.00	1.00	1.00	**0.00**	1.00	1.00	1.00	**0.00**	1.00	1.00	1.00	**0.00**	**0.00**	**0.00**	1.00	1.00	1.00
	IL-17A	1.00	1.00	**0.00**	0.12	1.00	**0.00**	1.00	1.00	**0.00**	0.14	1.00	**0.00**	1.00	**0.00**	0.85	1.00	**0.01**	1.00	1.00	0.11	1.00	**0.01**	1.00	1.00	1.00	0.10	1.00	**0.02**
	IFN-γ	1.00	1.00	0.10	1.00	1.00	0.13	1.00	1.00	**0.00**	1.00	1.00	**0.02**	1.00	0.17	1.00	1.00	0.17	1.00	1.00	0.36	1.00	1.00	1.00	1.00	1.00	0.22	1.00	1.00
	TNF	1.00	1.00	**0.00**	**0.00**	1.00	**0.00**	1.00	1.00	**0.00**	**0.00**	1.00	**0.00**	1.00	**0.00**	**0.00**	1.00	**0.00**	1.00	1.00	1.00	1.00	**0.00**	0.12	1.00	**0.00**	0.06	1.00	**0.00**
	CD3+ T cells	1.00	1.00	0.13	1.00	0.63	1.00	0.32	1.00	1.00	1.00	**0.04**	1.00	1.00	1.00	1.00	0.07	1.00	1.00	1.00	**0.00**	1.00	1.00	0.45	1.00	1.00	0.15	**0.00**	1.00
	CD20+ B cells	0.37	1.00	0.68	1.00	0.24	1.00	1.00	0.95	1.00	1.00	**0.00**	1.00	1.00	1.00	1.00	0.19	1.00	1.00	1.00	**0.00**	1.00	1.00	0.33	1.00	1.00	0.09	0.05	1.00
	CD68+ macrophages	0.56	1.00	**0.00**	1.00	0.15	1.00	1.00	0.15	0.17	1.00	**0.00**	1.00	1.00	**0.00**	1.00	0.58	0.74	0.61	1.00	**0.00**	1.00	0.94	0.26	1.00	1.00	**0.01**	**0.01**	1.00
	Eosinophils	1.00	**0.00**	1.00	1.00	0.54	1.00	1.00	**0.00**	1.00	1.00	0.17	1.00	1.00	**0.00**	0.55	1.00	0.12	**0.00**	1.00	0.23	1.00	1.00	1.00	1.00	1.00	1.00	0.50	1.00
	Neutrophils	1.00	0.15	1.00	1.00	**0.00**	1.00	1.00	**0.00**	1.00	1.00	**0.00**	0.40	1.00	**0.01**	1.00	0.41	1.00	0.26	1.00	**0.00**	0.60	1.00	0.07	1.00	1.00	0.62	**0.00**	1.00
Bacterial community variables	OTU130_*Corynebacterium*	0.36	1.00	1.00	1.00	0.77	1.00	1.00	1.00	1.00	1.00	**0.00**	0.39	1.00	1.00	1.00	0.06	1.00	1.00	1.00	0.14	1.00	1.00	1.00	1.00	1.00	1.00	0.34	1.00
	OTU5_*Corynebacterium*	0.16	1.00	1.00	1.00	1.00	1.00	1.00	1.00	1.00	1.00	**0.01**	0.55	1.00	1.00	1.00	0.44	1.00	1.00	1.00	0.16	1.00	1.00	0.21	1.00	1.00	1.00	0.89	1.00
	OTU16_*Haemophilus*	1.00	0.30	1.00	1.00	1.00	1.00	1.00	1.00	1.00	1.00	1.00	1.00	0.63	1.00	1.00	0.10	1.00	**0.05**	1.00	0.68	1.00	0.44	1.00	1.00	1.00	1.00	1.00	1.00
	OTU2_*Streptococcus*	1.00	1.00	1.00	1.00	0.08	1.00	1.00	1.00	1.00	0.53	0.16	1.00	1.00	1.00	0.83	0.25	1.00	1.00	0.68	0.37	1.00	1.00	**0.01**	1.00	1.00	0.69	**0.02**	1.00
	OTU3_*Pseudomonas*	1.00	1.00	1.00	1.00	0.29	1.00	0.11	1.00	1.00	1.00	0.25	1.00	0.08	1.00	1.00	0.38	1.00	0.17	1.00	0.37	1.00	0.16	1.00	1.00	1.00	1.00	1.00	1.00
	OTU9_*Propionibacterium*	0.46	1.00	0.16	0.23	0.81	1.00	1.00	1.00	1.00	1.00	**0.01**	1.00	0.88	1.00	1.00	0.09	1.00	1.00	1.00	**0.00**	1.00	0.30	**0.01**	0.77	0.29	1.00	1.00	1.00
	OTU20_*Anaerococcus*	0.14	1.00	0.64	1.00	1.00	1.00	1.00	1.00	1.00	1.00	0.06	1.00	0.74	1.00	1.00	1.00	1.00	1.00	1.00	0.18	1.00	1.00	1.00	1.00	1.00	0.96	1.00	1.00
	Bacterial load	0.55	1.00	0.33	1.00	0.86	1.00	1.00	1.00	1.00	1.00	**0.01**	1.00	1.00	1.00	1.00	0.30	1.00	1.00	1.00	**0.01**	1.00	1.00	0.11	1.00	1.00	1.00	0.68	1.00

*Significant *p*-values (α = 0.05) are expressed in bold.
†Categorical variables are tested as binary (yes/no) using Fisher's exact test and continuous variables tested using Dunn's test of multiple comparisons, with Bonferroni adjustment for multiple comparisons.
‡Corticosteroids in the 4 weeks prior to surgery.
AERD = aspirin-exacerbated respiratory disease. SC = Subject cluster.

Supplementary Table 4. Probability of OTUs occurring in each subject cluster*

	OTUs	SC1	SC2	SC3	SC4	SC5	SC6	SC7	SC8	Analysis of deviance *p*-value**
		Subject clusters								
15 most abundant OTUs	OTU_1_*Staphylococcus*	1.00	1.00	0.80	1.00	1.00	1.00	1.00	0.86	0.260
	OTU_130_*Corynebacterium*	0.97	1.00	0.60	1.00	0.89	0.93	0.90	0.86	0.297
	OTU_5_*Corynebacterium*	1.00	1.00	0.60	1.00	1.00	0.93	0.90	0.57	**0.004**
	OTU_16_*Haemophilus*	0.30	0.23	0.20	0.50	0.39	0.07	0.70	0.57	**0.039**
	OTU_6_*Peptoniphilus*	0.83	0.92	0.80	0.75	0.67	0.79	0.80	0.43	0.355
	OTU_4_*Corynebacterium*	0.30	0.46	0.00	0.25	0.44	0.64	0.30	0.00	**0.016**
	OTU_2_*Streptococcus*	0.83	0.85	0.80	1.00	0.89	0.86	1.00	0.29	**0.026**
	OTU_8_*Moraxella*	0.13	0.31	0.20	0.50	0.33	0.36	0.20	0.14	0.544
	OTU_3_*Pseudomonas*	0.17	0.15	0.20	0.25	0.17	0.14	0.60	0.57	0.090
	OTU_9_*Propionibacterium*	0.97	1.00	0.60	1.00	0.67	0.93	0.80	0.29	**0.000**
	OTU_20_*Anaerococcus*	0.90	0.85	0.60	1.00	0.50	0.79	0.50	0.43	**0.008**
	OTU_7_*Dolosigranulum*	0.40	0.23	0.40	0.50	0.39	0.36	0.60	0.14	0.593
	OTU_14_*Anaerococcus*	0.80	0.85	0.60	0.75	0.67	0.79	0.60	0.43	0.500
	OTU_18_*Fusobacterium*	0.43	0.46	0.40	0.75	0.33	0.43	0.30	0.43	0.876
	OTU_19_*Finegoldia*	0.80	0.77	0.40	1.00	0.72	0.64	0.50	0.43	0.147
Other OTUs with significant analysis of deviance	OTU_23_*Enterobacteriaceae*	0.30	0.69	0.60	0.00	0.28	0.36	0.50	0.14	**0.048**
	OTU_112_*Haemophilus*	0.23	0.31	0.20	1.00	0.50	0.00	0.30	0.57	**0.001**
	OTU_1059_*Corynebacterium*	0.87	1.00	0.60	1.00	0.67	0.86	0.70	0.29	**0.004**
	OTU_1588_*Anaerococcus*	0.77	0.77	0.60	0.75	0.39	0.64	0.40	0.14	**0.017**
	OTU_33_*Prevotella*	0.20	0.23	0.00	0.75	0.17	0.07	0.00	0.00	**0.023**
	OTU_57_*Micrococcus*	0.43	0.77	0.20	1.00	0.44	0.29	0.50	0.14	**0.012**
	OTU_60_*Neisseria*	0.37	0.46	0.00	1.00	0.28	0.21	0.20	0.29	**0.025**
	OTU_51_*Enhydrobacter*	0.40	0.62	0.40	0.75	0.28	0.14	0.50	0.00	**0.016**
	OTU_73_*Prevotella*	0.37	0.38	0.00	0.00	0.33	0.14	0.00	0.14	**0.024**
	OTU_118_*Porphyromonas*	0.40	0.38	0.20	0.25	0.22	0.00	0.20	0.00	**0.019**
	OTU_141_*Bacteroides*	0.20	0.00	0.00	0.00	0.06	0.00	0.40	0.14	**0.020**
	OTU_297_*Novosphingobium*	0.17	0.15	0.00	0.25	0.00	0.29	0.00	0.00	**0.048**
	OTU_70_*Pasteurellaceae*	0.10	0.00	0.20	0.75	0.06	0.14	0.10	0.00	**0.038**
	OTU_1149_*Propionibacterium*	0.30	0.62	0.40	0.50	0.28	0.14	0.10	0.00	**0.029**
	OTU_185_*Subdoligranulum*	0.17	0.08	0.00	0.00	0.17	0.07	0.60	0.14	**0.038**
	OTU_144_*Blautia*	0.10	0.00	0.00	0.00	0.00	0.07	0.60	0.00	**0.001**
	OTU_204_*Akkermansia*	0.03	0.15	0.00	0.00	0.00	0.07	0.50	0.00	**0.006**
	OTU_230_*Prevotella*	0.17	0.31	0.00	0.00	0.00	0.00	0.00	0.00	**0.014**
	OTU_363_*Prevotella*	0.00	0.00	0.20	0.00	0.22	0.00	0.00	0.00	**0.028**
	OTU_803_*Chryseobacterium*	0.00	0.00	0.00	0.50	0.00	0.00	0.10	0.00	**0.036**

*Probability of occurrence for the 15 most abundant OTUs, and all other OTUs with significant analysis of deviance results, based on logistic regression modelling incorporating the presence/absence of each OTU in subject clusters. Probability values are color-coded from white (0.00) to grey (1.00).
**Differences in the presence/absence for each OTU across groups tested using analysis of deviance. Significant *p*-values (α = 0.05) are expressed in bold.
